# EphA2 Proteolytic Fragment as a Sensitive Diagnostic Biomarker for Very Early-stage Pancreatic Ductal Carcinoma

**DOI:** 10.1158/2767-9764.CRC-23-0087

**Published:** 2023-09-15

**Authors:** Shinya Sato, Masatoshi Nakagawa, Takeshi Terashima, Soichiro Morinaga, Yohei Miyagi, Eisaku Yoshida, Toru Yoshimura, Motoharu Seiki, Shuichi Kaneko, Makoto Ueno, Taro Yamashita, Naohiko Koshikawa

**Affiliations:** 1 Molecular Pathology and Genetics Division, Kanagawa Cancer Center Research Institute, Yokohama, Japan.; 2 Department of Pathology, Kanagawa Cancer Center Hospital, Yokohama, Japan.; 3 Morphological Analysis Laboratory, Kanagawa Cancer Center Research Institute, Yokohama, Japan.; 4 Research and Development, Abbott Japan LLC, Chiba, Japan.; 5 Department of Life Science and Technology, Tokyo Institute of Technology, Yokohama, Japan.; 6 Advanced Preventive Medical Sciences Research Center, Kanazawa University Hospital, Kanazawa, Japan.; 7 Department of Gastroenterological Surgery, Kanagawa Cancer Center Hospital, Yokohama, Japan.; 8 Graduate School of Medical Sciences, Kanazawa University, Kanazawa, Japan.; 9 Department of Gastroenterology, Kanagawa Cancer Center Hospital, Yokohama, Japan.; 10 Clinical Cancer Proteomics Laboratory, Kanagawa Cancer Center Research Institute, Yokohama, Japan.

## Abstract

Cleavage of erythropoietin-producing hepatocellular ephrin receptor A2 (EphA2) triggers malignant progression and yields an N-terminal fragment (EphA2-NF) detectable in sera from patients with pancreatic ductal carcinoma. We established a quantitative automated chemiluminescence immunoassay for EphA2-NF and evaluated serum EphA2-NF levels as a biomarker to diagnose pancreatic ductal carcinoma in the test and validation cohorts. The EphA2-NF value was elevated (above the cutoff: mean ± SD) in more than half of the patients with stage I/II pancreatic ductal carcinoma. Among patients receiving standard chemotherapy for pancreatic ductal carcinoma [gemcitabine plus nab-paclitaxel (GnP)], the median survival time of patients with elevated serum EphA2-NF was half that of patients with values below the cutoff. Patients with intraductal papillary mucinous neoplasm (IPMN), a precancerous pancreatic ductal carcinoma lesion, also show high serum EphA2 levels, which are associated with an increase in pancreatic duct size and the development of pancreatic ductal carcinoma in some cases. IHC showed loss of EphA2-NF staining in IPMN with pancreatic ductal carcinoma, but not in the normal epithelium or IPMN without pancreatic ductal carcinoma, regardless of the histologic grade. These results suggest that EphA2 cleavage is an essential event that occurs very early in pancreatic ductal carcinoma development, and that the consequent release of EphA2-NF can be detected in the serum. Thus, serum EphA2-NF could be a diagnostic biomarker for very early-stage pancreatic ductal carcinoma and pancreatic ductal carcinoma development from high-risk IPMN and as a prognostic biomarker after chemotherapy with GnP.

**Significance:**

EphA2 N-terminus deletion is involved in pancreatic ductal carcinoma development from high-risk IPMN and EphA2-NF produced by cleavage can be used as a serum biomarker to diagnose pancreatic ductal carcinoma and predict pancreatic ductal carcinoma development from high-risk IPMN.

## Introduction

Erythropoietin-producing hepatocellular ephrin receptor A2 (EphA2) is the largest member of the receptor tyrosine kinase family, with at least 14 receptors and eight ligands identified in vertebrates (1, 2). EphA2 is weakly expressed in various normal epithelial cells, and its ligands (membrane-bound ephrin-A1–A5) are also expressed in adjacent epithelial cells under physiologic conditions (3). Stimulation of EphA2 by its ligands induces phosphorylation of tyrosine-588 residues (pY^588^-EphA2), which function as tumor suppressors. This diminishes excess EGFR signaling in normal epithelial cells, thereby maintaining a stable epithelial structure and function (3, 4). However, EphA2 is also highly expressed in various cancers, and its expression level correlates with cancer malignancy and prognosis (3). In the absence of ligand stimulation, the EGFR/MAPK/PI3K pathway phosphorylates the serine-897 residue of EphA2 (pS^897^-EphA2, termed ligand-independent oncogenic signaling) to promote cell proliferation, survival, motility, and chemoresistance through the activation of RhoG/RACK signaling pathways (5, 6) ultimately stimulating malignant tumor progression.

Several studies using cultured cells have demonstrated that EphA2 overexpression increases tumor cell growth, motility, and metastasis (3, 7) and expression of EphA2 alone in human mammary epithelial MCF10A cells is sufficient to confer tumorigenicity in mice (8). Therefore, EphA2 is closely involved in the processes involved in the transition from tumor carcinogenesis to malignant progression, suggesting a potential therapeutic target for cancer (7, 9).

EphA2 ligands (ephrin-A) are expressed in several types of cancer cells (10, 11). Moreover, several cancer cells release the soluble form of EphA2-As, which can induce pY^588^-EphA2 expression in brain, breast, and cervical cancer (12, 13). Although, the mechanism by which cancer cells escape ligand-dependent tumor-suppressive signaling *in vivo* remains unclear, we have recently elucidated a novel molecular mechanism that regulates the conflicting cellular functions of EphA2 signaling (14). Proteolytic cleavage of EphA2 by membrane type 1 matrix metalloproteinase (MT1-MMP) on the surface of cancer cells converts EphA2 from a ligand-bound to a ligand-unbound form in many cancer types (14–18). MT1-MMP is a membrane protein that is upregulated in cancer cells and plays an important role in various cancerous events from carcinogenesis to malignant progression (7, 9, 19, 20). Because cleaved EphA2 acts as a transducer to promote oncogenic signaling, even in the presence of ephrin-A ligands, it could be a valuable target for molecular cancer therapeutics. Furthermore, the N-terminal fragment of EphA2 (EphA2-NF), released by MT1-MMP cleavage, has been detected in commercially available cancer sera, suggesting its potential as a diagnostic biomarker for several cancer types (18, 21). A sandwich ELISA showed that serum EphA2-NF levels were significantly higher in most pancreatic cancers than in other cancers, suggesting its potential as a specific biomarker for pancreatic cancer (21).

Pancreatic cancer is caused by mutations in oncogenes and tumor suppressor genes (22) and is a highly fatal disease, ranking as the fourth leading cause of cancer-related deaths in the United States in 2019 (23). The 5-year survival rate at the time of diagnosis is 8.5%, with approximately 80% of patients presenting with unresectable or metastatic disease. Detecting early-stage pancreatic cancer using conventional diagnostic techniques remains challenging (24–27). Furthermore, there are no effective drugs for the treatment of pancreatic cancer, and early detection and medical intervention offer hope for a cure (24). Conventional serum tumor markers for pancreatic carcinoma, such as carcinoembryonic antigen (CEA) and carbohydrate antigen 19-9 (CA19-9), are not sufficiently accurate for early diagnosis (28). Furthermore, CA19-9 levels do not increase in Lewis antigen-negative cases (∼10% of patients with pancreatic cancer in Japan; ref. 29). Therefore, new diagnostic biomarker candidates have been developed, such as apolipoprotein A isoforms (apoA2-i) and circular RNAs, that can detect not only stage I/II pancreatic cancer but also benign precancerous diseases such as intraductal papillary mucinous neoplasms (IPMN; refs. 30, 31). Nevertheless, there remains an urgent need to develop more accurate and effective diagnostic biomarkers for early-stage pancreatic cancer.

In this study, we established a serum EphA2-NF quantitative analysis system, which was used to evaluate the potential of EphA2-NF as a new biomarker for early pancreatic cancer diagnosis and prediction of therapy response using sera from independent testing and validation cohorts. Furthermore, we evaluated serum EphA2-NF level as a predictive biomarker for pancreatic cancer development in patients with IPMN. In addition to the clinical evaluation of serum EphA2-NF, the *in vivo* state of EphA2 cleavage was detected using IHC staining of surgically resected tissues from patients with IPMN.

## Materials and Methods

### Antigens and Antibodies

**FIGURE 1:**
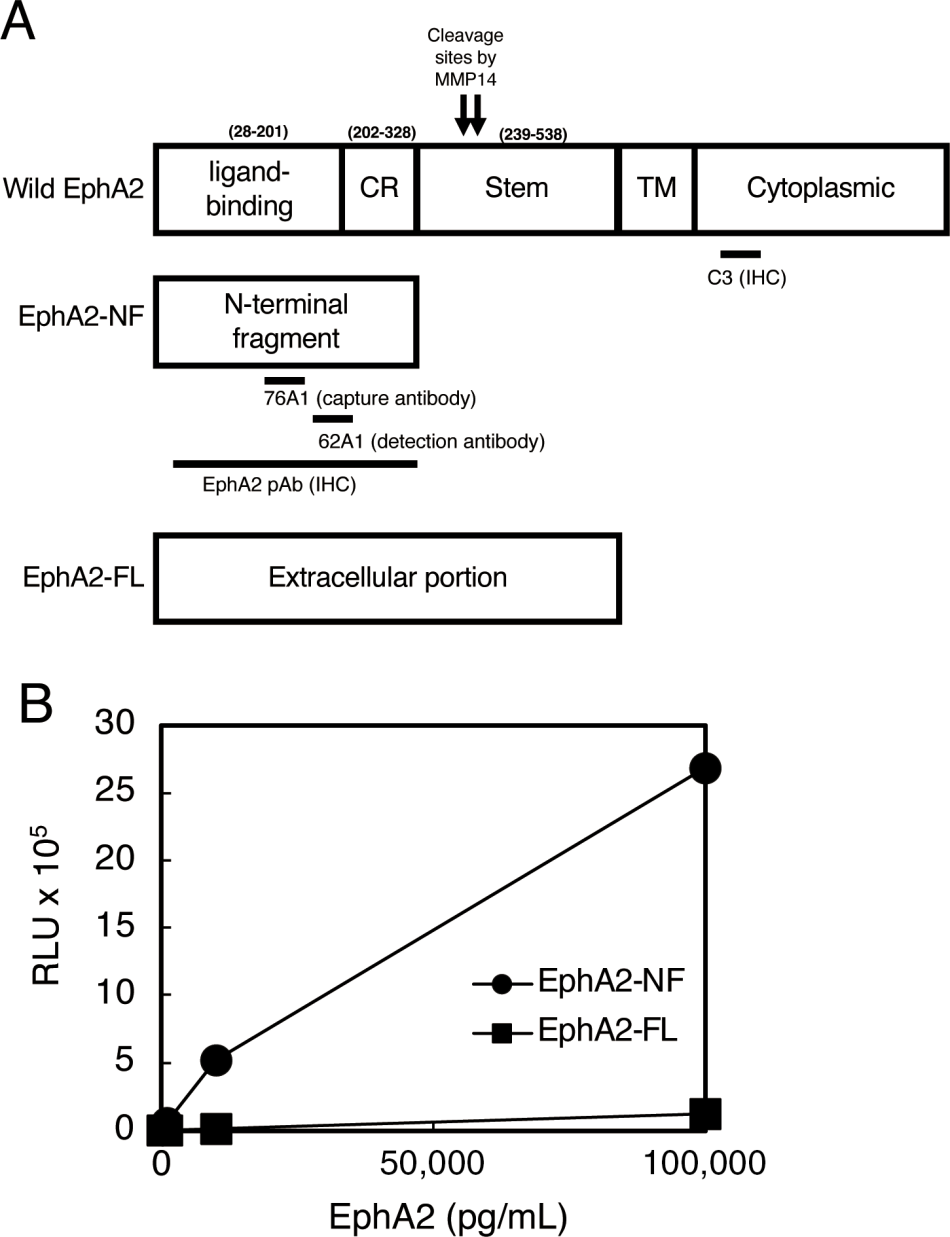
Antigens and antibody epitopes used in the CLIA. **A,** Schematic of EphA2 molecules. Wild-type EphA2 is an intact form that contains a transmembrane (TM) cytoplasmic domain. EphA2-NF is an N-terminal fragment released by MT1-MMP processing. EphA2-FL (full-length) is an extracellular form without the TM and cytoplasmic domains. Epitopes of the EphA2 antibodies are shown. SP, ligand-binding signal peptide; CR, cysteine-rich. **B,** Standard curves of EphA2-NF (circles) and EphA2-FL (squares) for the CLIA using mAbs against 76A1 and 62A1. The detection range of EphA2-NF was 10–10^5^ pg/mL, while the detection of EphA2-FL was negligible (squares).

Antibody epitopes are shown in Fig. 1A. EphA2-NF (amino acids 28–328) released from the cell surface by MT1-MMP cleavage and the full-length extracellular form (EphA2-FL; amino acids 28–537) were prepared as reported previously (Fig. 1A). Mouse anti-EphA2 mAbs 76A1 and 62A1 were prepared as reported previously and used for an automated chemiluminescent immunoassay (CLIA; ref. 21). For IHC, anti-EphA2 C-terminus mAb (C3) was purchased from Santa Cruz Biotechnology. Rabbit anti-EphA2 N-terminus (amino acids 28–328) pAb was prepared in our laboratory (Supplementary Fig. S1). Antigens used to prepare the antibodies are shown in Fig. 1A.

### Quantitative Measurement of Serum EphA2-NF in Patients with Pancreatic Diseases Using the CLIA

76A1 mAb was immobilized on magnetic microparticles using carboxylic acid groups, and 62A1 mAb was labeled with acridinium. A two-step sandwich assay was performed for a fully automated CLIA using ARCHITECT (Abbott Laboratories). Recombinant EphA2-NF protein was diluted in PBS containing 1% (w/v) BSA, 0.1% (v/v) Tween-20, and 0.1% (v/v) ProClin 300, which was used as the test standard at concentrations of 0, 10, 20, 50, 100, 1000, 10,000, and 100,000 pg/mL (Fig. 1B). The detailed analytic conditions are described in a previous report (32).

### Clinical Serum and Tissue Samples

The clinical study protocol was approved by the Ethics Boards of Kanazawa University Hospital (2297–7), Kanagawa Cancer Center (2018-EKI-075, 2018-EKI-52), Abbott (#2020-001), and Tokyo Institute of Technology (#2020-026). After obtaining informed consent, serum samples were collected from both cohorts. Initially, we collected sera from 97 patients with pancreatic cancer, 6 patients with pancreatic neuroendocrine tumors (PNET), 46 patients with IPMN, and 150 healthy donors (HD) at Kanazawa University Hospital, which were used as the test cohort to evaluate the performance of the assay and establish and confirm the potential utility of the serum EphA2-NF level as a biomarker of pancreatic cancer, especially in its early stages. To validate the usefulness of EphA2-NF as an early-stage pancreatic cancer biomarker, we collected sera at Kanagawa Cancer Center from 19 patients with PNET, 16 with IPMN (low-grade IPMN: 5, high-grade IPMN: 11), and 479 with pancreatic cancer. Serum EphA2-NF and CA19-9 levels were measured using the ARCHITECT. The validation cohort also included 89 surgically resected IPMN tissues (low-grade IPMN: 17, high-grade IPMN: 72): 34 with and 55 without pancreatic cancer. All tissue samples were obtained from the Kanagawa Cancer Center.

### IHC

IHC was performed using primary antibodies against EphA2. Primary antibodies against the EphA2 C-terminus (C3, diluted 1:1,000, mAb) and EphA2 N-terminus (diluted 1:300, rabbit pAb) were used for IHC. ImageJ software (version 1.52a, NIH, Bethesda, MD; ref. 33) was used to analyze the extent of staining. The average staining scores ranged from 5 to 8, indicating high staining intensity and positivity. The proportion and intensity of staining were assessed using the Allred score ratio (ASR), which is defined as the EphA2 N-terminus staining score divided by the EphA2 C-terminus staining score (34). Staining scores of the normal ductal epithelium and IPMN tissues were assessed in all cases. For cases with multiple lesions exhibiting different degrees of malignancy, such as IPMN and pancreatic cancer in the same specimen, we assessed the staining scores separately for each lesion.

### Statistical Analyses

Analyse-it version 5.40.2 (Analyse-it Software) was used to generate all scatter plots, ROC curves, and to calculate the area under the ROC curves (AUC). The AUC of the ROC curve indicates the diagnostic accuracy, and the ROC curve, plotted as the true-positive fraction versus the false-positive fraction with various threshold values, was used to analyze the diagnostic accuracy. The optimal cut-off point was calculated based on the mean value and SD of the HD group. Differences between the groups were tested using the Mann–Whitney *U* test to determine statistical significance; *P* < 0.05 was considered significant. Prism 8.4.3 (GraphPad Software) was used for Kaplan–Meier survival curve analyses. Cox proportional hazards models, analyzed using SPSS software (ver. 23.0; IBM Japan), were used to identify risk factors for hepatocellular carcinoma.

### Ethics Approval

The study was approved by the Institutional Review Board of the Kanazawa University Hospital (2297-7), Kanagawa Cancer Center (2018-EKI-075 and 2018-EKI-52), Abbott (#2020-001), and the Tokyo Institute of Technology (#2020-026). All patients agreed to participate in the study and provided written informed consent. All experiments using clinical samples were performed following the ethical principles of the Declaration of Helsinki.

### Data Availability Statement

All relevant data are within the article and its Supplementary Data.

## Results

### CLIA for EphA2-NF

In our previous study, a sandwich ELISA with 76A1 and 62A1 mAbs was used to measure EphA2-NF values in cancer sera, and the detection range was approximately 10–250 pg/mL (21). To improve the detection range, sensitivity, accuracy, reproducibility, and throughput, we developed an automated CLIA. The detection range for diluted recombinant EphA2-NF protein in the buffer solution (0–10^5^ pg/mL) was 10–10^5^ pg/mL (Fig. 1B). The recovery rates in the four normal specimens were in the range of 81.7%–90.3% for samples spiked with 50 pg/mL EphA2-NF and 85.8%–111% for samples spiked with 1,000 pg/mL EphA2-NF. These ranges are acceptable for clinical diagnosis. CLIA could specifically detect the processed and released form (EphA2-NF) of EphA2 by MT1-MMP cleavage. CLIA did not detect the presence of recombinant EphA2-FL protein in the buffer.

### Evaluation of Serum EphA2-NF as a Biomarker of Pancreatic Cancer in the Test Cohort

**FIGURE 2:**
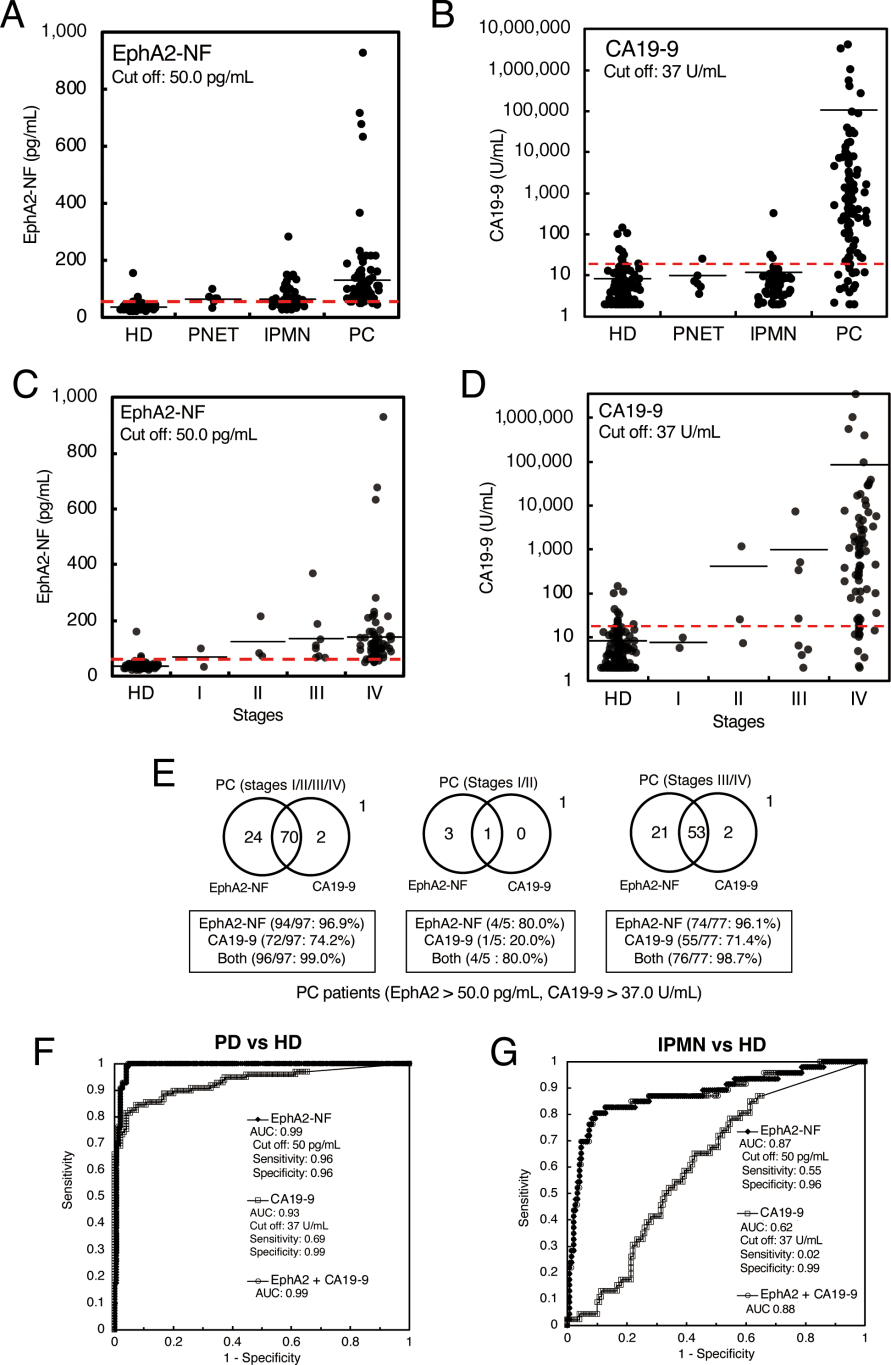
Diagnostic performance of EphA2-NF and CA19-9 for pancreatic cancer in the test cohort. Dot plots of serum EphA2-NF (**A**) and CA19-9 (**B**) levels from 150 HDs, and 6 patients with PNETs, 46 patients with IPMNs, and 97 patients with pancreatic cancer in the test cohort. The cut-off value for EphA2-NF was defined at 50.0 pg/mL (mean + 1 SD of HDs). The cut-off value of CA19-9 was set at 37 U/mL. Dot plots of EphA2-NF (**C**) and CA19-9 (**D**) levels in pancreatic cancer sera from the test cohort by stage. In this analysis, 82 cases of pancreatic cancer with staging information were utilized. **E,** Venn diagrams of EphA2-NF and CA19-9 in patient sera with pancreatic cancer (left, stage I/II/III/IV), early-stage pancreatic cancer (center, stage I/II), and late-stage pancreatic cancer (right, stage III/IV). **F,** ROC curves of serum EphA2-NF and CA19-9 for differentiating between pancreatic cancer and HDs. **G,** ROC curve of serum EphA2-NF and CA19-9 for differentiating IPMN and HDs.

To determine the clinical utility of serum EphA2-NF for pancreatic cancer diagnosis, we first measured its concentration using CLIA in 299 serum samples in the test cohort (Fig. 2A; Supplementary Table S1). A summary of serum EphA2-NF values and detailed clinical information of the patients are presented in Table 1A. The mean values for the serum EphA2-NF level were 131.0 pg/mL for pancreatic cancer cases (*n* = 97), 68.2 pg/mL for IPMNs (*n* = 6), 65.5 pg/mL for PNETs (*n* = 66), and 36.0 pg/mL for HDs (*n* = 150). The SD values for EphA2-NF in the PNET and HD groups were small and tightly clustered around the mean values. In contrast, the distribution of EphA2-NF values in patients with pancreatic cancer was significantly wider than that in patients with benign disease and HD. The cut-off values for EphA2-NF were 50.0 and 74.0 pg/mL (based on the means ± SD for HD vs. pancreatic cancer and for HDs + IPMN vs. pancreatic cancer, respectively). In addition, the cut-off values for EphA2-NF calculated by Youden index were 45.5 and 59.8 pg/mL for HD versus pancreatic cancer and HD + IPMN versus pancreatic cancer, respectively (Supplementary Fig. S2). Because the cut-off values for EphA2-NF by Youden index and mean ± SD were similar, we used 50 pg/mL as the cut-off value for EphA2-NF based on the mean ± SD in this study.

**TABLE 1A:** Comparison of clinical background in the test cohort (Kanazawa University)

		EphA2 < 50 pg/mL (*n* = 24)	EphA2 ≧ 50 pg/mL (*n* = 125)	*P*
PNET and IPMN (*n* = 52)	Age (year)	68^a^	72^a^	0.270^b^
	Gender (male/female)	8/13	19/12	0.100^c^
	CA19-9 (U/mL)	5.0^a^	6.0^a^	0.389^b^
PC (*n* = 97)	Age (year)	57^a^	67^a^	0.160^b^
	Gender (male/female)	0/3	66/28	0.010^c^
	CA19-9 (U/mL)	258.6^a^	440.0^a^	0.739^b^

Abbreviation: PC, pancreatic cancer.

^a^Median.

^b^ *t* test.

^c^ *χ* ^2^ test.

The distribution of CA19-9 values in pancreatic cancer cases was wider than that in other groups (Fig. 2B). Interestingly, the serum EphA2-NF values of some IPMN cases were higher than the cut-off value (Fig. 2A), which was not the case for CA19-9. After confirming the prognosis, some EphA2-NF–high cases showed pancreatic cancer development and an increase in pancreatic duct size. Indeed, the correlation between the EphA2-NF value and pancreatic duct size was analyzed in patients with IPMN, including invasive carcinoma cases. Cases with a high level of EphA2-NF had a tendency to exhibit an enlarged pancreatic duct size as shown in Supplementary Fig S3. Furthermore, serum EphA2-NF levels tended to increase in a stage-dependent manner (Fig. 2C; Supplementary Table S2). EphA2-NF showed a lower cut-off value for stage I pancreatic cancer, although this trend was not observed for CA19-9 (Fig. 2D). These results indicate that serum EphA2 may be a novel diagnostic and predictive biomarker for early-stage pancreatic cancer and pancreatic cancer development from IPMN, although a lower number of early-stage pancreatic cancer cases were evaluated in the test than in the validation cohort.

Using a cut-off value of 50.0 pg/mL for EphA2-NF and 37 U/mL for CA19-9, EphA2-NF and CA19-9 detected 96.9% (94/97) and 74.2% (72/97) of patients with pancreatic cancer (stages I/II/III/IV), 80.0% (4/5) and 20.0% (1/5) of patients with early-stage pancreatic cancer (stages I and II), and 96.1% (74/77) and 71.4% (55/77) of patients with late-stage pancreatic cancer (stage III/IV), respectively (Fig. 2E). Furthermore, the combination of EphA2 and CA19-9 detected 96/97 (99.0%) patients with pancreatic cancer, 4/5 (80.0%) patients with early-stage pancreatic cancer, and 76/77 (98.7%) patients with late-stage pancreatic cancer. These results showed that the combination of EphA2 and CA19-9 appeared to be relatively more capable of detecting earlier stages of pancreatic cancer than CA19-9 alone.

The AUC values for pancreatic cancer diagnosis by serum EphA2-NF or CA19-9 (vs. HDs) were 0.99 and 0.93, respectively, suggesting that serum EphA2-NF was superior to CA19-9 for pancreatic cancer diagnosis (Fig. 2F). To confirm whether pancreatic cancer carcinogenesis can be detected by serum EphA2-NF, we performed ROC analysis using IPMN sera, including very early-stage pancreatic cancer that may not be detected by imaging tests because pancreatic cancer develops annually in only approximately 1% of IPMN cases. The AUC values for accurately diagnosing IPMN were 0.88 and 0.61 with EphA2-NF and CA19-9, respectively (Fig. 2G). Therefore, serum EphA2-NF appears to be a suitable molecule for IPMN diagnosis.

### Confirmation of Serum EphA2-NF as an Early-stage Pancreatic Cancer Biomarker in the Validation Cohort

**FIGURE 3:**
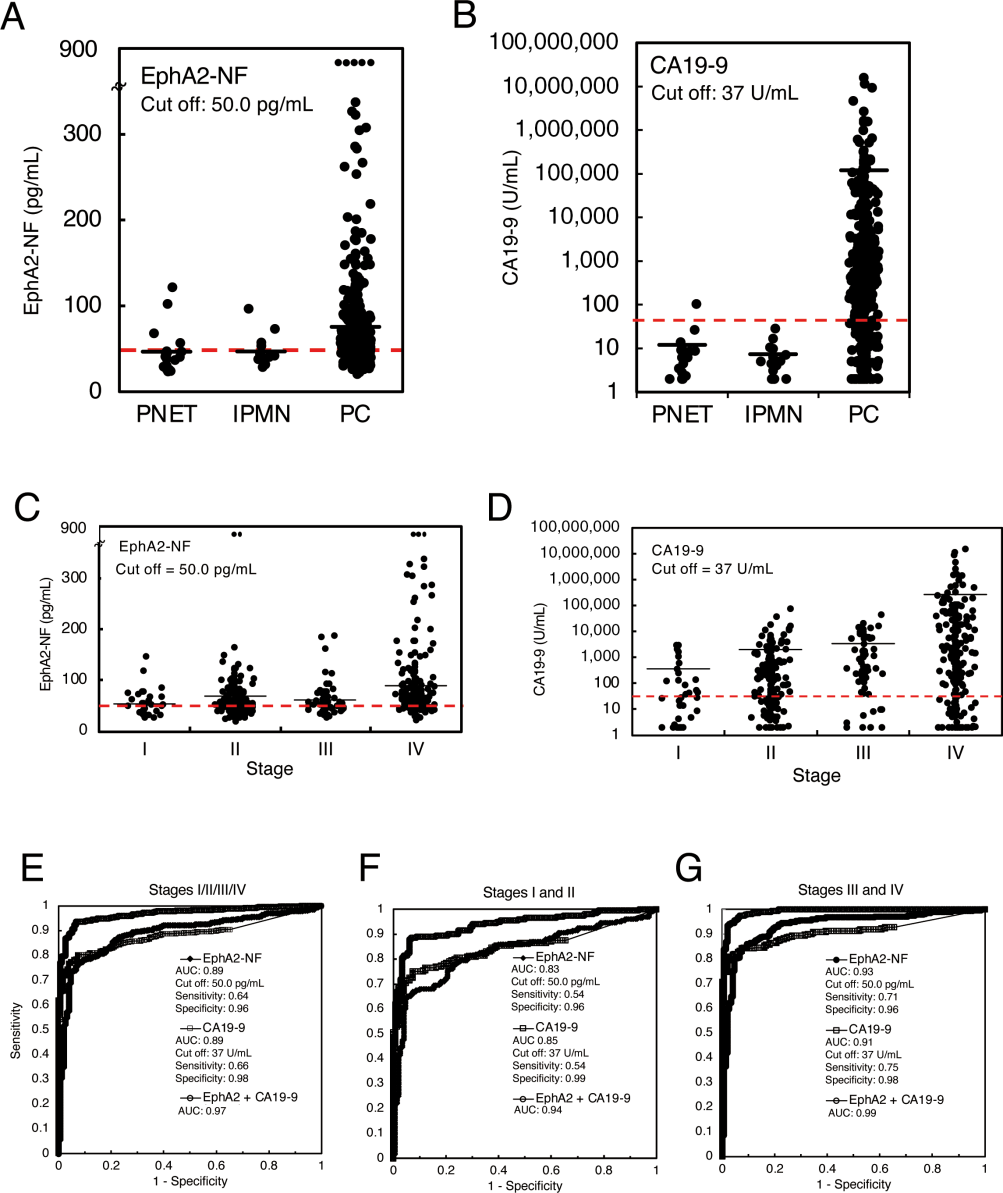
Diagnostic performance of EphA2-NF and CA19-9 for pancreatic cancer in the validation cohort. Dot plots of serum EphA2-NF (**A**) and CA19-9 (**B**) in the validation cohort of patients with pancreatic cancer (*n* = 472), IPMNs (*n* = 16), and PNETs (*n* = 19). The cut-off value for EphA2-NF and CA19-9 was defined as 50.0 pg/mL and 37 U/mL, respectively, from the test cohort analysis. Dot plots of EphA2-NF (**C**) and CA19-9 (**D**) in pancreatic cancer sera from the validation cohort by stage. **E**–**G,** ROC curves of serum EphA2-NF and CA19-9 levels for differentiating pancreatic cancer (left, stage I/II/III/IV), early-stage pancreatic cancer (center, stage I/II), late-stage pancreatic cancer (right, stage III/IV), and HD.

The mean values for serum EphA2-NF in the validation cohort were 75.6 pg/mL for pancreatic cancer cases (*n* = 472), 46.9 pg/mL for IPMN (*n* = 16), and 46.6 pg/mL for PNET (*n* = 19; Fig. 3A; Supplementary Table S3). A summary of serum EphA2-NF values and detailed clinical information of the patients are presented in Table 1B. The SD values for EphA2-NF in IPMN and PNET were small and tightly clustered around the mean, whereas the distribution of EphA2-NF values in pancreatic cancer cases was significantly wider than in patients with benign disease. Similar to the EphA2-NF values, the distribution of CA19-9 values in pancreatic cancer cases was wider than that in the other groups (Fig. 3B). The mean values of serum EphA2-NF and CA19-9 were above the cut-off values for stages I and II pancreatic cancer (Fig. 3C and D; Supplementary Table S4), similar to the test cohort. Moreover, because there were lower numbers of IPMN and PNET in the validation cohort than pancreatic cancer, these cases in the test cohort were combined (IPMN: 25, PNET: 25) and analyzed by using dot blot (Supplementary Fig. S4), yielding similar results.

**TABLE 1B:** Comparison of clinical background in the validation cohort (Kanagawa Cancer Center)

		EphA2 < 50 pg/mL (*n* = 195)	EphA2 ≧ 50 pg/mL (*n* = 312)	*P*
PNET and IPMN (*n* = 35)	Age (year)	60^a^	73^a^	0.004^b^
	Gender (male/female)	9/17	7/2	0.025^c^
	CA19-9 (U/mL)	6.6^a^	5.0	0.316^b^
PC (*n* = 472)	Age (year)	65^a^	71^a^	<0.001^b^
	Gender (male/female)	85/84	187/116	0.016^c^
	Stage (I/II/III/IV)	18/73/26/52	18/91/37/157	<0.001^c^
	Treatment (resection/chemotherapy)^d^	91/73	105/183	<0.001^c^
	CA19-9 (U/mL)	259.7^a^	623.9^a^	0.066^b^

Abbreviation: PC, pancreatic cancer.

^a^Median.

^b^ *t* test.

^c^ *χ* ^2^ test.

^d^Treatment including GnP, mFFX (oxaliplatin, irinotecan, and 5-fluorouracil), S-1 plus GnP, gemcitabine alone, gemcitabine plus S-1, S-1 plus 5-fluorouracil, S-1, irinotecan and oxaliplatin, and S-1 plus radiation. Treatment options: GnP(Gem/nab-PTX): *n* = 208, Gemox (S-1 + GnP): *n* = 1, mFFX (mFOLFIRINOX): *n* = 26, Gem (Gemcitabine alone): *n* = 11, GS (Gem/S-1): *n* = 2, S-1: *n* = 3, S-1 + 5-FU: *n* = 1, S-IROX (JCOC1611): *n* = 1, S-1 + RT: *n* = 1.

The AUCs for the diagnosis of pancreatic cancer (stages I/II/III/IV) using serum EphA2-NF, CA19-9, or EphA2-NF + CA19-9 (vs. test cohort HDs) in the validation cohort were 0.89, 0.89, and 0.97, respectively (Fig. 3E). Furthermore, the AUCs using EphA2-NF, CA19-9, or EphA2-NF + CA19-9 were 0.83, 0.85, and 0.94, respectively, for early-stage pancreatic cancer (stage I/II; Fig. 3F), and 0.93, 0.91, and 0.99, respectively, for late-stage pancreatic cancer (stage III/IV; Fig. 3G). These results demonstrated that serum EphA2-NF has sufficient diagnostic potential as a biomarker for early-stage pancreatic cancer. Furthermore, the combination of EphA2-NF and CA19-9 can improve the diagnostic accuracy of early-stage pancreatic cancer.

### EphA2 and CA19-9 are Independent Serum Biomarkers for Pancreatic Cancer

**FIGURE 4:**
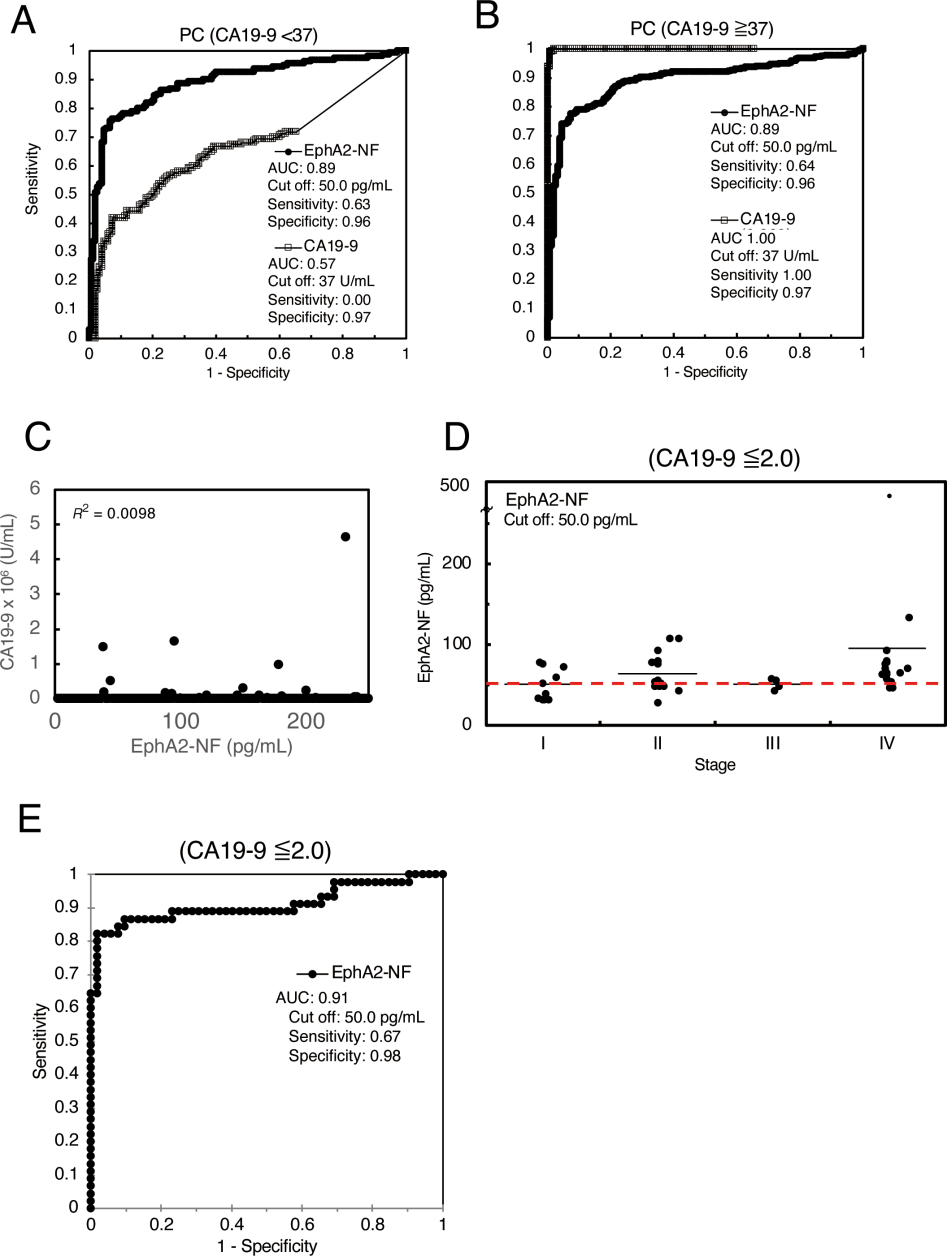
EphA2-NF is an independent diagnostic biomarker for pancreatic cancer from CA19-9. **A** and **B,** The ROC curves of serum EphA2-NF and CA19-9 to differentiate pancreatic cancer and HDs divided into high (A) and low (B) CA19-9 groups. The AUC for the diagnosis of pancreatic cancer by serum EphA2-NF (vs. test cohort HDs) for CA19-9 high or low was 0.87. **C,** There was no significant correlation between serum EphA2-NF and CA19-9 levels. **D,** Dot plot analysis of serum EphA2-NF in CA19-9–negative patients. **E,** ROC curves of serum EphA2-NF for pancreatic cancer with CA19-9 ≤2.0 U/mL.

To investigate whether serum EphA2-NF could complement CA19-9, ROC curve analysis was performed after dividing the patients into two groups: CA19-9–high and –low (Fig. 4A and B). In the validation cohort including patients with CA19-9–high (≥37.0 U/mL) pancreatic cancer, the ROC curve had a high AUC value of 1.00 (Fig. 4B). The AUC for pancreatic cancer diagnosis by serum EphA2-NF (vs. test cohort HDs) in the CA19-9–low (<37.0 U/mL) group was 0.89, suggesting that serum EphA2-NF has excellent diagnostic ability regardless of the CA19-9 level. Notably, when positive and negative rates for EphA2-NF and CA19-9 were examined in early and late-stage pancreatic cancers, approximately 20% of CA19-9–low pancreatic cancer (<37 U/mL) cases exceeded the EphA2-NF cutoff (≥50 pg/mL; Supplementary Fig. S5), and no significant correlation was found between EphA2-NF and CA19-9 levels (Fig. 4C).

We further examined whether serum EphA2 level could be used to diagnose pancreatic cancer in patients with a very low CA19-9 level (≤2.0 U/mL). All mean values of patients with stage I/II/III/IV pancreatic cancer exceeded the cut-off value, and the diagnostic tendency was similar to that of the entire patient cohort (Fig. 4D; Supplementary Table S5). Furthermore, serum EphA2-NF showed a higher AUC of 0.91 (cutoff: 50 pg/mL, sensitivity: 0.67, specificity: 0.98) for negative cases (CA19-9 ≤ 2.0 U/mL; Fig. 4E). These results indicate that serum EphA2-NF could be a useful biomarker for CA19-9–negative case including Lewis antigen-negative patients.

### Overall Survival of Patients with Pancreatic Cancer in the Serum EphA2-NF–high and –low Groups

**FIGURE 5:**
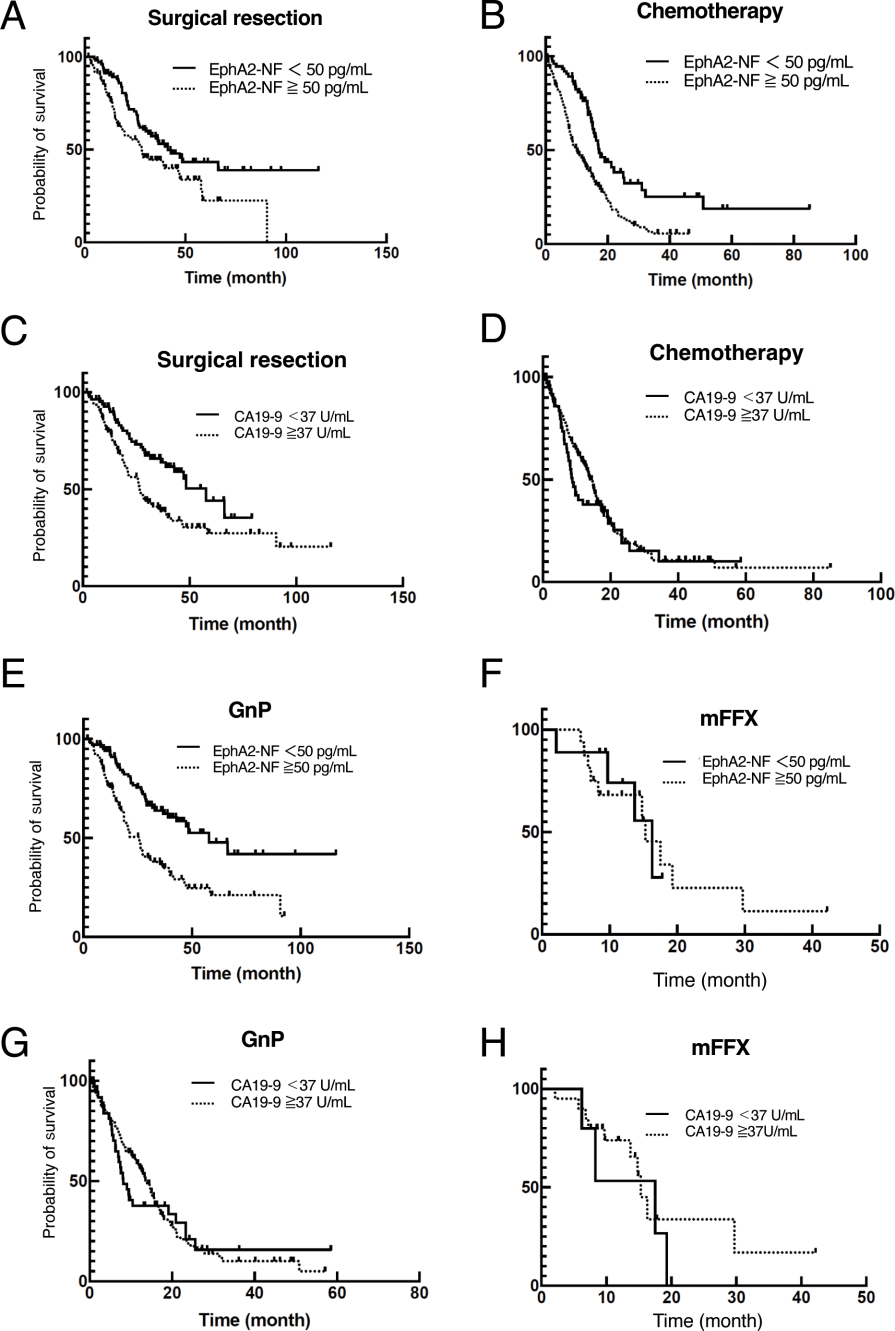
Clinical analysis of serum EphA2-NF levels for patients with pancreatic cancer treated with surgical resection or chemotherapy. Kaplan–Meier analyses of patients with pancreatic cancer treated with surgical resection (**A**) and chemotherapy (**B**) after classification into serum EphA2-NF–high (≥50 pg/mL) and –low (<50 pg/mL) groups. Kaplan–Meier analyses of patients with pancreatic cancer treated with surgical resection (**C**) and chemotherapy (**D**) classified into CA19-9–high (≥37 U/mL) and –low (<37 U/mL) groups. Kaplan–Meier analyses of patients with pancreatic cancer treated with GnP (**E**) and modified FOLFIRINOX (oxaliplatin, irinotecan, and 5-fluorouracil, mFFX) (**F**) after classification into serum EphA2-NF (≥50 pg/mL) and low (<50 pg/mL) groups. Kaplan–Meier analyses of patients with pancreatic cancer treated with GnP (**G**) and mFFX (**F**) classified into CA19-9–high (≥37 U/mL) and –low (<37 U/mL) groups.

Patients with pancreatic cancer in the validation cohort were classified into the serum EphA2-NF–high (≥50 pg/mL) and -low (<50 pg/mL) groups. When comparing the overall survival (OS) of patients who underwent surgical resection (Fig. 5A; Supplementary Table S6) or standard chemotherapy (Fig. 5B; Supplementary Table S6) using the Kaplan–Meier method, patients with EphA2-NF–low had significantly longer OS than those with EphA2-NF–high in both treatment groups. We also compared OS between the CA19-9–high (≥37 U/mL) and –low (<37 U/mL) groups treated with surgical resection and chemotherapy, showing that patients with CA19-9–low also had longer OS than those with CA19-9–high after surgical resection (Fig. 5C; Supplementary Table S7), but not after chemotherapy (Fig. 5D; Supplementary Table S7). To further analyze the OS between the EphA2-NF–high and –low groups treated with chemotherapy, we divided the patients according to two standard chemotherapy regimens: gemcitabine and nab-paclitaxel (GnP) and modified FOLFIRINOX (oxaliplatin, irinotecan, and 5-fluorouracil, mFFX; Fig. 5E and F; Supplementary Table S8). The serum EphA2-NF–high group had a lower median survival time (MST; ∼9.5) than the EphA2-NF–low group (∼17.2 months) among those receiving GnP treatment but not for those receiving the mFFX regimen (Fig. 5E and F). We further compared OS between the CA19-9–high (≥37 U/mL) and –low (<37 U/mL) groups treated with surgical resection and chemotherapy, showing that patients with CA19-9–low also had longer OS than those with CA19-9–high after surgical resection (Fig. 5C). In contrast to the results for EphA2-NF, there was no significant difference in OS between the CA19-9–high and –low groups of patients receiving chemotherapy (Fig. 5G and H; Supplementary Table S9). We also performed univariate Cox regression analyses to identify prognostic factors for patients with pancreatic cancer and found that stages, treatments, CA19-9, and EphA2-NF were associated with a significantly poor prognosis of pancreatic cancer (Table 2). A multivariate Cox regression analysis indicated that the significant independent prognostic factors for pancreatic cancer were treatments [HR, 0.62; 95% confidence interval (CI), 0.41–0.95; *P* = 0.03], stages (HR, 1.95; 95% CI, 1.29–2.96; *P* < 0.01), and EphA2-NF (HR, 1.97; 95% CI, 1.52–2.56; *P* < 0.01; Table 2). These data suggest that serum EphA2-NF, but not CA19-9 status, in patients with pancreatic cancer might be associated with chemosensitivity to GnP treatment.

**TABLE 2:** Univariate and multivariate Cox regression analyses of prognostic factor of pancreatic cancer

	Univariate		Multivariate	
Variables	HR (95% CI)	*P*	HR (95% CI)	*P*
Age (≧60 vs. <60 years)	1.19 (0.90–1.57)	0.23	1.04 (0.76–1.35)	0.93
Sex (male vs. female)	1.18 (0.94–1.48)	0.16	1.02 (0.81–1.29)	0.88
Stages (III/IV vs. I/II)	3.20 (2.50–4.10)	<0.01	1.95 (1.29–2.96)	<0.01
Treatments (surgical recession vs. Chemotherapy)	0.32 (0.25–0.41)	<0.01	0.62 (0.41–0.95)	0.03
EphA2 (≧50 vs. <50 pg/mL)	2.30 (1.80–3.00)	<0.01	1.97 (1.52–2.56)	<0.01
CA19-9 (≧37 vs. <37 U/mL)	1.63 (1.25–2.13)	<0.01	1.23 (0.94–1.63)	0.14

### IHC of EphA2 N- and C-terminus Expression in IPMN

**FIGURE 6:**
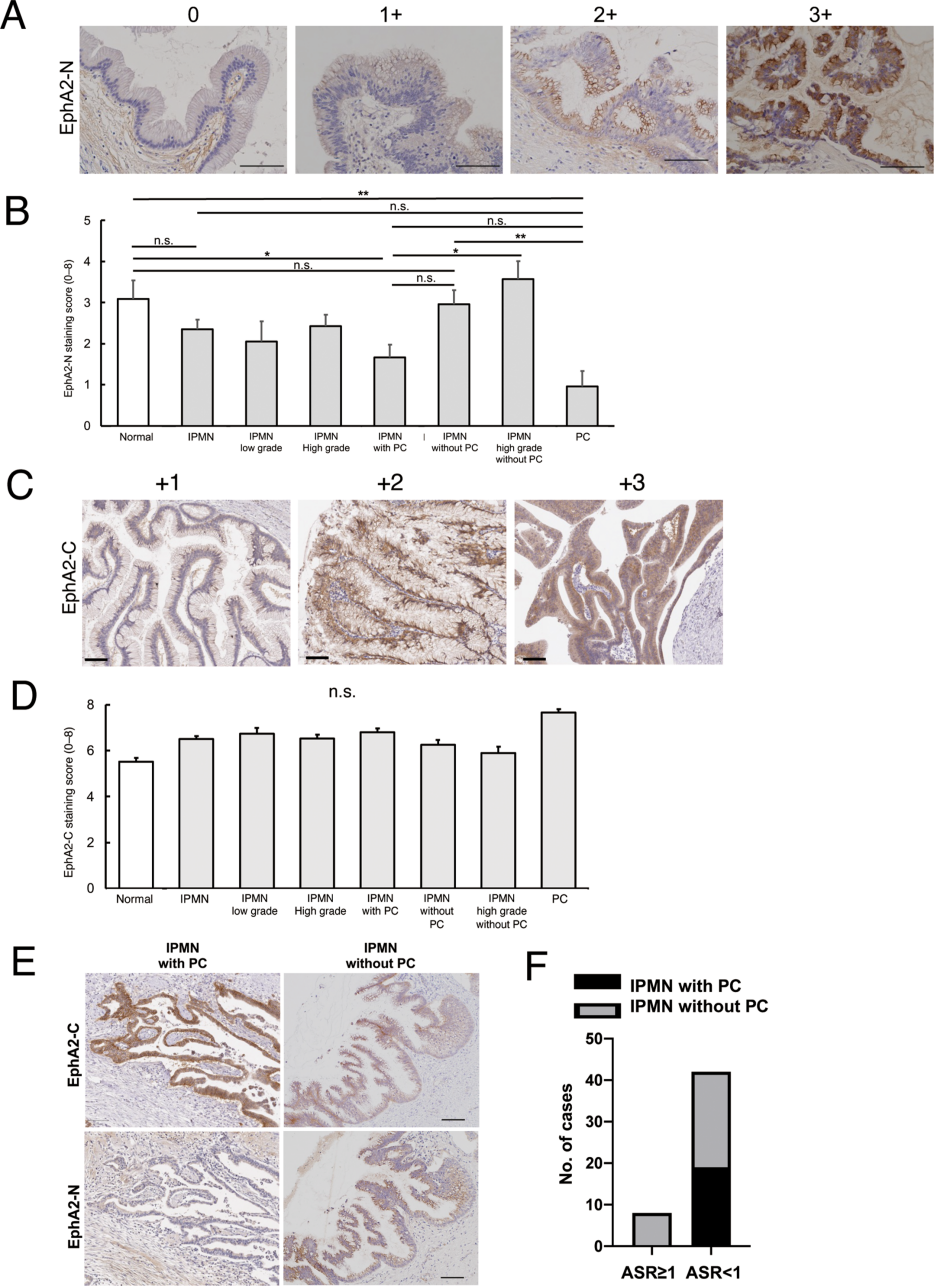
IHC of the EphA2 cleavage state in IPMN with and without pancreatic cancer. **A,** Representative images of IPMN cells stained for the EphA2 N-terminus. 0, no staining; 1+, weakly positive staining; 2+, moderately positive staining; 3+, strongly positive staining. Scale bar: 100 μm. **B,** EphA2 N-terminus staining scores in normal (*n* = 67), IPMN (*n* = 89), low-grade IPMN (*n* = 19), high-grade IPMN (*n* = 70), IPMN with pancreatic cancer (*n* = 42), IPMN without pancreatic cancer (*n* = 47), high-grade IPMN without pancreatic cancer (*n* = 42), and pancreatic cancer (*n* = 25). ns, no significance; *, *P* < 0.05; **, *P* < 0.01; ***, *P* < 0.001. **C,** Representative images of IPMN tissues stained for the EphA2 C-terminus. 0, no staining; 1+, weakly positive staining; 2+, moderately positive staining; 3+, strongly positive staining. Scale bar: 100 μm. **D,** EphA2 C-terminus staining scores in normal (*n* = 67), IPMN (*n* = 89), low-grade IPMN (*n* = 19), high-grade IPMN (*n* = 70), IPMN with pancreatic cancer (*n* = 42), IPMN without pancreatic cancer (*n* = 47), high-grade IPMN without pancreatic cancer (*n* = 42), and pancreatic cancer (*n* = 25). **E,** Representative images of IPMN with or without pancreatic cancer stained for the EphA2 N- or C-terminus. Scale bar: 50 μm. **F,** Statistical analysis of the ASR of IPMN with and without pancreatic cancer. The *χ*^2^ test was performed. n.s., no significance.

To analyze the state of EphA2 cleavage *in vivo*, IHC for EphA2 N- and C-termini was performed in the normal duct epithelium, IPMN with and without pancreatic cancer, and pancreatic cancer tissues (Fig. 6A and B). The EphA2 N-terminus staining score was significantly reduced in IPMN with pancreatic cancer compared with that in the normal duct epithelium, whereas there was no significant difference in staining between IPMN without pancreatic cancer and the normal duct epithelium (Fig. 6B). Furthermore, the EphA2 N-terminus staining score was significantly reduced in pancreatic cancer compared with that in IPMN without pancreatic cancer, and was also reduced compared with that in high-grade IPMN without pancreatic cancer. However, there were no significant differences in EphA2 C-terminus staining between normal ductal epithelium, low-grade IPMN, high-grade IPMN, IPMN with pancreatic cancer, IPMN without pancreatic cancer, and high-grade IPMN with pancreatic cancer, even when examining multiple lesions with different malignancies in the same specimen separately (Fig. 6C and D).

Finally, we combined the results of the expression levels of EphA2 N- and EphA2 C-termini in IPMN with and without pancreatic cancer according to the ASR (Fig. 6E). The frequency of pancreatic cancer coexistence in IPMN cases with ASR <1 was significantly higher than in cases with ASR ≥1 (Fig. 6F, Supplementary Table S10). Because IPMN cases with an ASR <1 may be more likely to develop pancreatic cancer in the future, we will continue to monitor these patients.

## Discussion

Because early diagnosis and treatment have the potential to completely cure pancreatic cancer, the development of innovative biomarkers that can detect this disease in its early stages is an urgent issue (25, 35). In this study, we used automated CLIA to show that the level of EphA2-NF, produced by the proteolytic cleavage of EphA2, was elevated in patient sera from early-stage pancreatic cancer and at pancreatic cancer development from IPMN. We also showed by IHC *in vivo* that EphA2 cleavage occurs at the N-terminus of pancreatic cancer and IPMN with pancreatic cancer by IHC *in vivo*.

Compared with CA19-9, as the conventional tumor marker for pancreatic cancer, serum EphA2-NF could diagnose approximately 50% of early-stage pancreatic cancer cases in both the test and validation cohorts. Except for early-stage pancreatic cancer, which can be incidentally diagnosed by imaging tests such as CT and MRI, most patients with pancreatic cancer are diagnosed at a late stage. Therefore, EphA2-NF may aid in the diagnosis of early-stage pancreatic cancer, and future studies should evaluate the use of EphA2-NF together with imaging modalities.

We also found that serum-EphA2-NF could effectively predict the prognosis after chemotherapy for pancreatic cancer, which was not the case for CA19-9. When comparing the OS obtained from patients under standard regimens of pancreatic cancer chemotherapy with GnP and mFFX, the MST among EphA2-NF–low cases was approximately two times longer than that of EphA2-NF–high cases treated with GnP. In contrast, there was no correlation between serum EphA2-NF levels and clinical outcomes of patients undergoing mFFX treatment. Therefore, EphA2 cleavage may be specifically involved in the chemoresistance to gemcitabine and/or nab-paclitaxel in GnP treatment. A recent study demonstrated that exosome-mediated EphA2 may be required for the acquisition of gemcitabine resistance in pancreatic cancer cells (36). Previous reports have shown that ligand-independent EphA2 signaling (pS^897^-EphA2) induced by RSK1/2 and/or AKT signaling is a trigger for pancreatic cancer chemoresistance, and the N-terminal truncated EphA2 fragment can be a transmitter at the cancer cell surface for pS^897^-EphA2 signaling (7, 37). These findings suggest that patients with locally advanced pancreatic cancer may be stratified according to their predicted response to GnP by measuring the serum EphA2 level.

In the test cohort, EphA2-NF levels were higher in IPMN sera than in HDs, offering a new serum biomarker for detecting IPMN, which is currently only possible by imaging. Importantly, follow-up of EphA2-NF–high IPMN cases confirmed the development of pancreatic cancer and pancreatic duct dilatation. Slight dilatation of the main pancreatic duct is a marker of a high risk of pancreatic cancer (38). Therefore, serum EphA2-NF could be used to detect a high-risk case of pancreatic cancer development from IPMN. In addition, IHC analysis of IPMN cases with and without pancreatic cancer showed that all tissues with pancreatic cancer had a defect in the N-terminus of EphA2, which was detected in both high-grade and some low-grade IPMN cases, suggesting that EphA2 cleavage occurred regardless of histologic grade. Although further detailed examination is required, the present results suggest that pancreatic cancer development does not depend on the tissue grade but is already determined at the onset of IPMN.

Importantly, serum EphA2-NF can improve pancreatic cancer serodiagnosis when combined with CA19-9 levels. Although CA19-9 is widely used for pancreatic cancer diagnosis, 10%–15% of patients are negative for Lewis A antigen, making it difficult to detect CA19-9 (39). Therefore, other serum biomarkers for pancreatic cancer, such as SPan-1, CA50, CA242, Dupan-2, and CEA, are used clinically (40, 41) even although their sensitivities and specificities are insufficient, and false-positive rates are as high as 20%–30%. Therefore, the diagnosis of Lewis antigen-negative pancreatic cancer must be based on diagnostic imaging. However, we found that serum EphA2-NF was a diagnostic biomarker independent of CA19-9, which was detected in more than half of the stage I/II pancreatic cancer cases that were CA19-9 negative (≤2.0 U/mL) and had a higher AUC of 0.91. On the basis of our results, serum EphA2-NF is considered to have sufficient performance for the diagnosis of Lewis-negative pancreatic cancer, for which CA19-9 is not applicable. Therefore, serum EphA2-NF, as a new biomarker for pancreatic cancer diagnosis independent of CA19-9, may improve the diagnostic accuracy in patients with Lewis-negative pancreatic cancer combined with imaging tests.

To date, a variety of innovative biomarkers, including EphA2, have been developed for early-stage pancreatic cancer diagnosis. Exosome-derived EphA2 has been suggested as a potential serum biomarker for pancreatic cancer diagnosis (42). In addition, because MT1-MMP, the enzyme responsible for EphA2 cleavage, is also present on the surface of exosomes (43), exosome EphA2 may be a source of EphA2-NF. Moreover, ELISA using large-scale clinical cohort samples enables measurement of plasma apoA2-ATQ/AT levels to identify high-risk cases, including pancreatic cancer and IPMN (44, 45). Although apoA2-ATQ/AT can diagnose pancreatic cancer in the early stages or pancreatic cancer development from IPMN, the CLIA for EphA2-NF is superior to that of apoA2-ATQ/AT.

Although many biomarker candidates with better diagnostic accuracy than CA19-9 have been identified in basic research studies, additional clinical research is needed to validate their utility. In the future, combining these innovative serum biomarkers for pancreatic cancer diagnosis at an early stage, such as through health checks, may improve pancreatic cancer therapeutic responses by enhancing the possibility of treating pancreatic cancer at an early stage.

This study demonstrated the clinical application of serum EphA2 as a biomarker for patients with early-stage pancreatic cancer using retrospective specimens. We are therefore planning a multicentered prospective clinical study to fully demonstrate the clinical usefulness of EphA2-NF in the near future. In addition, because we did not mention the molecular mechanisms by which EphA2-NF is involved in the development of pancreatic cancer in this study, we plan to conduct basic research using molecular biological analysis.

In summary, these results suggest that EphA2 N-terminus deletion is involved in pancreatic cancer development from IPMN and that EphA2-NF produced by cleavage can be used as a biomarker to diagnose pancreatic cancer and predict pancreatic cancer development from IPMN.

## Supplementary Material

Supplementary Fig. S1 (A) Schematic of recombinant EphA2-FL, EphA2-NF and stem portion. FLAG-tag was added at C-terminus. (B) These recombinant proteins were detected by anti-FLAG mAb and anti-EphA2-N-termius pAb.

Supplementary Fig. S2 Determination of cutoff value for CA19-9 and EphA2-NF. (A) PC vs HD and (B) PC vs IPMN + HD

Supplementary Fig. S3 Correlation between serum EphA2-NF and pancreatic duct size in IPMN including IPMC.

Supplementary Fig. S4 Dot blot analysis of PNET, IPMN and PC cases combined with test and validation cohorts. It became clear that serum EphA2-NF over the cutoff value existed in the benign pancreatic disease sera.

Supplementary Fig. S5 Comparison for the positive and negative ratios of CA19-9 and/or EphA2-NF in early-stage and late-stage PCs.

Supplementary Table S1 Serum EphA2-NF and CA19-9 levels from 150 healthy donors (HDs), and six patients with pancreatic neuroendocrine tumors (PNETs), 46 patients with intraductal papillary mucinous neoplasms (IPMNs), and 97 PC patients in the test cohort. The cutoff value for EphA2-NF was defined at 50.0 pg / ml (mean + 1 SD of HDs). The cutoff value of CA19-9 was set at 37 U / ml.

Supplementary Table S2 Serum EphA2-NF and CA19-9 levels in PC sera from the test cohort by stage. In this analysis, 82 cases of PC with staging information were utilized in addition to 150 healthy donors (HDs). The cutoff value for EphA2-NF was defined at 50.0 pg / ml (mean + 1 SD of HDs). The cutoff value of CA19-9 was set at 37 U / ml.

Supplementary Table S3 Serum EphA2-NF and CA19-9 in the validation cohort of patients with PC (n = 472), intraductal papillary mucinous neoplasms (IPMNs) (n = 16), and pancreatic neuroendocrine tumors (PNETs) (n = 19). The cutoff value for EphA2-NF and CA19-9 was defined as 50.0 pg / ml and 100 U / ml, respectively, from the test cohort analysis.

Supplementary Table S4 Serum EphA2-NF and CA19-9 in PC sera from the validation cohort by stage.

Supplementary Table S5 Serum EphA2-NF in CA19-9-negative patients with different stages.

Supplementary Table S6 Median survival time of PC patients treated with surgical resection and chemotherapy after classification into serum EphA2-NF high (≥50 pg / ml) and low (<50 pg / ml) groups.

Supplementary Table S7 Median survival time of PC patients treated with surgical resection and chemotherapy after classification into serum CA19-9 high (≥37 U / ml) and low (<37 U / ml) groups.

Supplementary Table S8 Median survival time of PC patients treated with gemcitabine and nab-paclitaxel (GnP) and modified FOLFIRINOX (oxaliplatin, irinotecan, and 5-fluorouracil, mFFX) after classification into serum EphA2-NF high (≥50 pg / ml) and low (<50 pg / ml) groups.

Supplementary Table S9 Median survival time of PC patients treated with GnP (G) and mFFX (F) classified into CA19-9 high (≥37 U / ml) and low (<37 U / ml) groups.

Supplementary Table S10 Statistical analysis of the Allred score ratio (ASR) of IPMN with and without PC. The chi-square test was performed.
