# Supplementary material for: EphA2 Proteolytic Fragment as a Sensitive Diagnostic Biomarker for Very Early-stage Pancreatic Ductal Carcinoma
Source: Cancer Res Commun. 2023 Sep 15;3(9):1862–74. doi: 10.1158/2767-9764.CRC-23-0087 (PMC10503484; doi:10.1158/2767-9764.CRC-23-0087)
Supplement: Supplementary Fig. S1 — (A) Schematic of recombinant EphA2-FL, EphA2-NF and stem portion. FLAG-tag was added at C-terminus. (B) These recombinant proteins were detected by anti-FLAG mAb and anti-EphA2-N-termius pAb. [file crc-23-0087-s01.pdf]

Fig S1

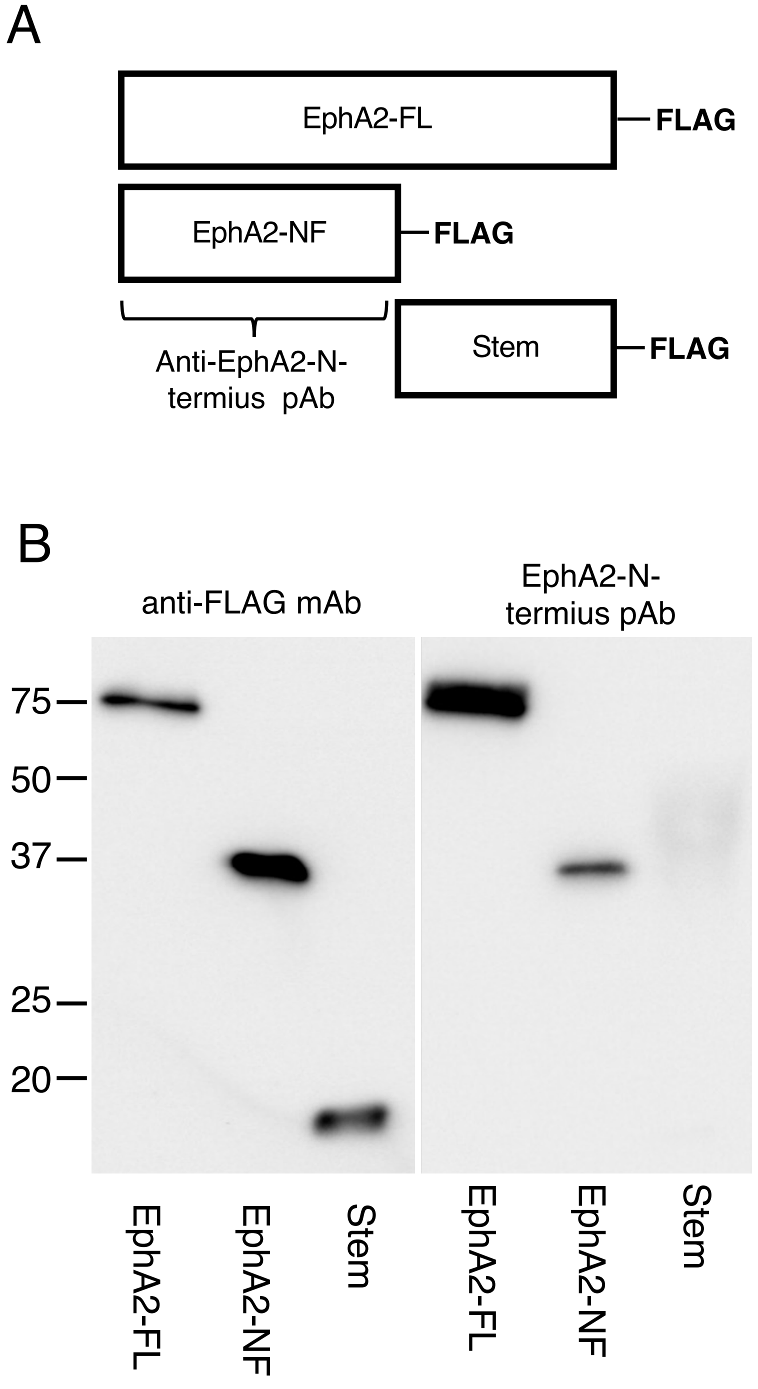

Figure S1.
(A) Schematic of recombinant EphA2-FL, EphA2-NF and stem portion. FLAG-tag was added at C-terminus. (B) These recombinant proteins were detected by anti-FLAG mAb and anti-EphA2-N-termius pAb.
