# Supplementary material for: EphA2 Proteolytic Fragment as a Sensitive Diagnostic Biomarker for Very Early-stage Pancreatic Ductal Carcinoma
Source: Cancer Res Commun. 2023 Sep 15;3(9):1862–74. doi: 10.1158/2767-9764.CRC-23-0087 (PMC10503484; doi:10.1158/2767-9764.CRC-23-0087)
Supplement: Supplementary Fig. S2 — Determination of cutoff value for CA19-9 and EphA2-NF. (A) PC vs HD and (B) PC vs IPMN + HD [file crc-23-0087-s02.pdf]

Fig S2

(A) PC vs HD

Cut off (Mean + SD)

	Cut off	PPV	NPV	TPF (Sensitivity)	FPF (1-Specificity)
EphA2-NF	48.4	0.94	0.98	0.96	0.96
CA19-9	25.9	0.94	0.85	0.74	0.97

Youden's index

	Cut off	PPV	NPV	TPF (Sensitivity)	FPF (1-Specificity)
EphA2-NF	45.5	0.93	1.00	1.00	0.95
CA19-9	24.8	0.93	0.89	0.81	0.96

(B) PC vs IPMN + HD

Cut off (Mean + SD)

Marker	Cut off	PPV	NPV	TPF (Sensitivity)	FPF (1-Specificity)
EphA2-NF	74.1	0.84	0.86	0.69	0.94
CA19-9	37.0	0.92	0.89	0.74	0.97

Youden's index

Marker	Cut off	PPV	NPV	TPF (Sensitivity)	FPF (1-Specificity)
EphA2-NF	59.8	0.8	0.93	0.86	0.9
CA19-9	25.6	0.8	0.96	0.8	0.96

Figure S2.
Determination of cutoff value for CA19-9 and EphA2-NF. (A) PC vs HD and (B) PC vs IPMN + HD
