# Supplementary material for: EphA2 Proteolytic Fragment as a Sensitive Diagnostic Biomarker for Very Early-stage Pancreatic Ductal Carcinoma
Source: Cancer Res Commun. 2023 Sep 15;3(9):1862–74. doi: 10.1158/2767-9764.CRC-23-0087 (PMC10503484; doi:10.1158/2767-9764.CRC-23-0087)
Supplement: Supplementary Fig. S3 — Correlation between serum EphA2-NF and pancreatic duct size in IPMN including IPMC. [file crc-23-0087-s03.pdf]

Fig S3

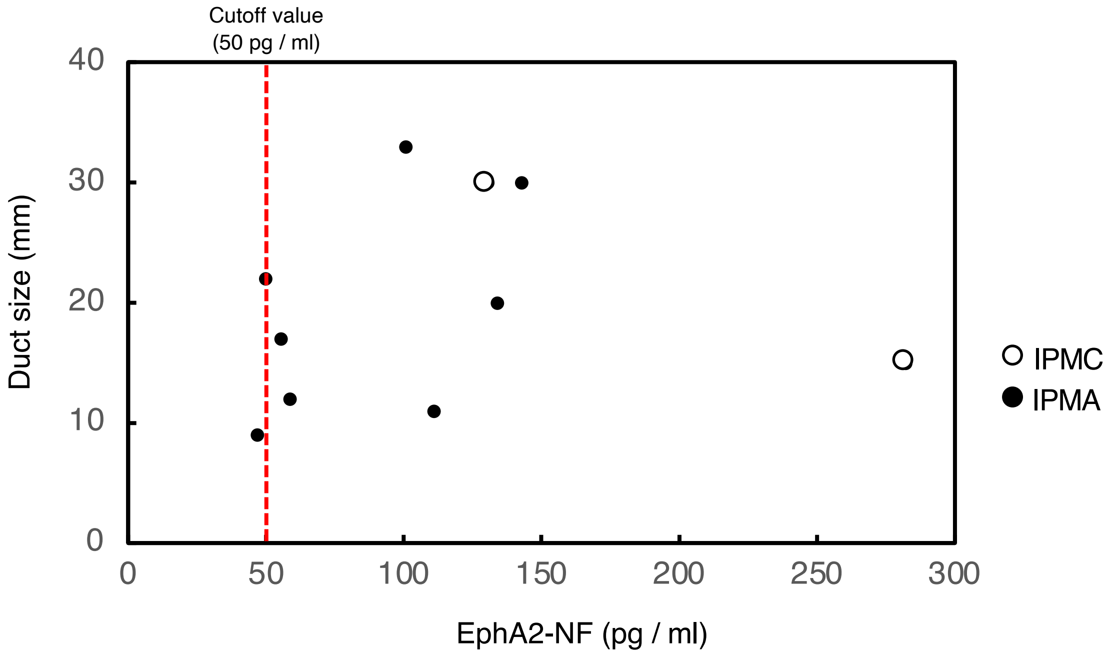

Figure S3.
Correlation between serum EphA2-NF and pancreatic duct size in IPMN including IPMC.
