# Supplementary material for: EphA2 Proteolytic Fragment as a Sensitive Diagnostic Biomarker for Very Early-stage Pancreatic Ductal Carcinoma
Source: Cancer Res Commun. 2023 Sep 15;3(9):1862–74. doi: 10.1158/2767-9764.CRC-23-0087 (PMC10503484; doi:10.1158/2767-9764.CRC-23-0087)
Supplement: Supplementary Fig. S4 — Dot blot analysis of PNET, IPMN and PC cases combined with test and validation cohorts. It became clear that serum EphA2-NF over the cutoff value existed in the benign pancreatic disease sera. [file crc-23-0087-s04.pdf]

Fig S4

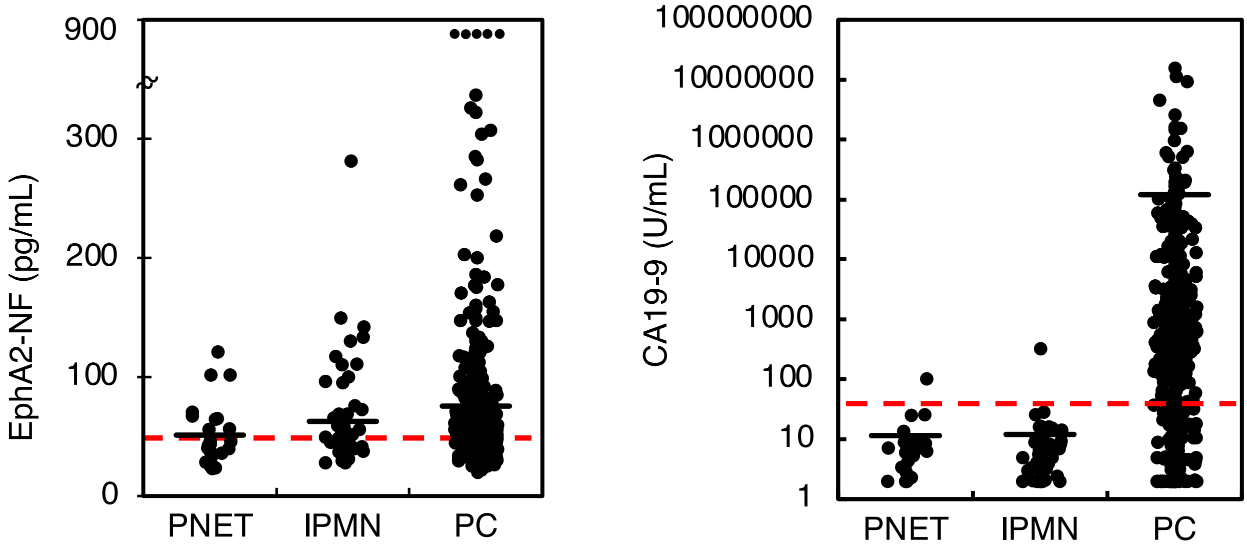

	N	EphA2-NF		CA19-9	
		mean	SD	mean	SD
PNET	25	51.2	25.9	11.5	20.0
IPMN	62	62.7	40.9	12.0	41.5
PC	472	75.6	74.6	120422.9	1029305.2

Figure S4.
Dot blot analysis of PNET, IPMN and PC cases combined with test and validation cohorts. It became clear that serum EphA2-NF over the cutoff value existed in the benign pancreatic disease sera.
