# Supplementary material for: EphA2 Proteolytic Fragment as a Sensitive Diagnostic Biomarker for Very Early-stage Pancreatic Ductal Carcinoma
Source: Cancer Res Commun. 2023 Sep 15;3(9):1862–74. doi: 10.1158/2767-9764.CRC-23-0087 (PMC10503484; doi:10.1158/2767-9764.CRC-23-0087)
Supplement: Supplementary Fig. S5 — Comparison for the positive and negative ratios of CA19-9 and/or EphA2-NF in early-stage and late-stage PCs. [file crc-23-0087-s05.pdf]

Fig S5

Stage I/II

	EphA2-NF ≧50	EphA2-NF <50
CA19-9 ≧ 37	35.0	29.0
CA19-9 < 37	19.5	16.5

Stage III/IV

	EphA2-NF ≧50	EphA2-NF <50
CA19-9 ≧ 37	56.6	24.3
CA19-9 < 37	14.7	4.4

Figure S5.
Comparison for the positive and negative ratios of CA19-9 and/or EphA2-NF in early-stage and late-stage PCs.
