# Supplementary material for: EphA2 Proteolytic Fragment as a Sensitive Diagnostic Biomarker for Very Early-stage Pancreatic Ductal Carcinoma
Source: Cancer Res Commun. 2023 Sep 15;3(9):1862–74. doi: 10.1158/2767-9764.CRC-23-0087 (PMC10503484; doi:10.1158/2767-9764.CRC-23-0087)
Supplement: Supplementary Table S1 — Serum EphA2-NF and CA19-9 levels from 150 healthy donors (HDs), and six patients with pancreatic neuroendocrine tumors (PNETs), 46 patients with intraductal papillary mucinous neoplasms (IPMNs), and 97 PC patients in the test cohort. The cutoff value for EphA2-NF was defined at 50.0 pg / ml (mean + 1 SD of HDs). The cutoff value of CA19-9 was set at 37 U / ml. [file crc-23-0087-s06.pdf]

Supplementary Table S1

	N	EphA2-NF		CA19-9	
		mean	SD	mean	SD
HD	150	36.0	12.4	8.4	17.6
PNET	6	65.5	22.1	9.7	8.1
IPMN	46	68.2	45.2	13.6	48.0
PC	97	131.0	139.2	107908.9	563960.1

Serum EphA2-NF and CA19-9 levels from 150 healthy donors (HDs), and six patients with pancreatic neuroendocrine tumors (PNETs), 46 patients with intraductal papillary mucinous neoplasms (IPMNs), and 97 PC patients in the test cohort. The cutoff value for EphA2-NF was defined at 50.0 pg / ml (mean + 1 SD of HDs). The cutoff value of CA19-9 was set at 37 U / ml.
