# Supplementary material for: EphA2 Proteolytic Fragment as a Sensitive Diagnostic Biomarker for Very Early-stage Pancreatic Ductal Carcinoma
Source: Cancer Res Commun. 2023 Sep 15;3(9):1862–74. doi: 10.1158/2767-9764.CRC-23-0087 (PMC10503484; doi:10.1158/2767-9764.CRC-23-0087)
Supplement: Supplementary Table S2 — Serum EphA2-NF and CA19-9 levels in PC sera from the test cohort by stage. In this analysis, 82 cases of PC with staging information were utilized in addition to 150 healthy donors (HDs). The cutoff value for EphA2-NF was defined at 50.0 pg / ml (mean + 1 SD of HDs). The cutoff value of CA19-9 was set at 37 U / ml. [file crc-23-0087-s07.pdf]

Supplementary Table S2

	N	EphA2-NF		CA19-9	
		mean	SD	mean	SD
HD	150	36.0	12.4	8.4	17.6
I	2	66.9	46.7	7.7	2.9
II	3	123.4	80.9	409.7	681.1
III	8	138.0	101.2	1014.4	2513.4
IV	69	138.1	142.5	84370.1	436717.5

Serum EphA2-NF and CA19-9 levels in PC sera from the test cohort by stage. In this analysis, 82 cases of PC with staging information were utilized in addition to 150 healthy donors (HDs). The cutoff value for EphA2-NF was defined at 50.0 pg / ml (mean + 1 SD of HDs). The cutoff value of CA19-9 was set at 37 U / ml.
