# Supplementary material for: EphA2 Proteolytic Fragment as a Sensitive Diagnostic Biomarker for Very Early-stage Pancreatic Ductal Carcinoma
Source: Cancer Res Commun. 2023 Sep 15;3(9):1862–74. doi: 10.1158/2767-9764.CRC-23-0087 (PMC10503484; doi:10.1158/2767-9764.CRC-23-0087)
Supplement: Supplementary Table S3 — Serum EphA2-NF and CA19-9 in the validation cohort of patients with PC (n = 472), intraductal papillary mucinous neoplasms (IPMNs) (n = 16), and pancreatic neuroendocrine tumors (PNETs) (n = 19). The cutoff value for EphA2-NF and CA19-9 was defined as 50.0 pg / ml and 100 U / ml, respectively, from the test cohort analysis. [file crc-23-0087-s08.pdf]

Supplementary Table S3

	N	EphA2-NF		CA19-9	
		mean	SD	mean	SD
PNET	19	46.6	25.8	12.1	22.7
IPMN	16	46.9	17.3	7.4	6.9
PC	472	75.6	74.6	120422.9	1029305.2

Serum EphA2-NF and CA19-9 in the validation cohort of patients with PC (n = 472), intraductal papillary mucinous neoplasms (IPMNs) (n = 16), and pancreatic neuroendocrine tumors (PNETs) (n = 19). The cutoff value for EphA2-NF and CA19-9 was defined as 50.0 pg / ml and 100 U / ml, respectively, from the test cohort analysis.
