# Supplementary material for: EphA2 Proteolytic Fragment as a Sensitive Diagnostic Biomarker for Very Early-stage Pancreatic Ductal Carcinoma
Source: Cancer Res Commun. 2023 Sep 15;3(9):1862–74. doi: 10.1158/2767-9764.CRC-23-0087 (PMC10503484; doi:10.1158/2767-9764.CRC-23-0087)
Supplement: Supplementary Table S4 — Serum EphA2-NF and CA19-9 in PC sera from the validation cohort by stage. [file crc-23-0087-s09.pdf]

Supplementary Table S4

Stage	N	EphA2-NF		CA19-9	
		mean	SD	mean	SD
I	36	53.1	25.6	361.3	802.2
II	164	67.8	86.1	1957.9	7242.8
III	63	62.1	31.9	3357.0	6961.5
IV	209	89.6	77.4	269349.4	1535914.8

Serum EphA2-NF and CA19-9 in PC sera from the validation cohort by stage.
