# Supplementary material for: EphA2 Proteolytic Fragment as a Sensitive Diagnostic Biomarker for Very Early-stage Pancreatic Ductal Carcinoma
Source: Cancer Res Commun. 2023 Sep 15;3(9):1862–74. doi: 10.1158/2767-9764.CRC-23-0087 (PMC10503484; doi:10.1158/2767-9764.CRC-23-0087)
Supplement: Supplementary Table S5 — Serum EphA2-NF in CA19-9-negative patients with different stages. [file crc-23-0087-s10.pdf]

Supplementary Table S5

Stage	N	EphA2-NF (pg/mL)	
		Mean	SD
I	10	50.9	19.3
II	15	65.3	23.9
III	4	51.4	6.4
IV	16	96.1	102.4

Serum EphA2-NF in CA19-9-negative patients with different stages.
