# Supplementary material for: EphA2 Proteolytic Fragment as a Sensitive Diagnostic Biomarker for Very Early-stage Pancreatic Ductal Carcinoma
Source: Cancer Res Commun. 2023 Sep 15;3(9):1862–74. doi: 10.1158/2767-9764.CRC-23-0087 (PMC10503484; doi:10.1158/2767-9764.CRC-23-0087)
Supplement: Supplementary Table S6 — Median survival time of PC patients treated with surgical resection and chemotherapy after classification into serum EphA2-NF high (≥50 pg / ml) and low (<50 pg / ml) groups. [file crc-23-0087-s11.pdf]

Supplementary Table S6

Median survival time (Month)

	EphA2-NF < 50 pg/mL	EphA2-NF ≧ 50	P value
Surgical resection	41.3	28.4	0.012
Chemotherapy	17.2	10.1	<0.001

Median survival time of PC patients treated with surgical resection and chemotherapy after classification into serum EphA2-NF high (≥50 pg / ml) and low (<50 pg / ml) groups.
