# Supplementary material for: EphA2 Proteolytic Fragment as a Sensitive Diagnostic Biomarker for Very Early-stage Pancreatic Ductal Carcinoma
Source: Cancer Res Commun. 2023 Sep 15;3(9):1862–74. doi: 10.1158/2767-9764.CRC-23-0087 (PMC10503484; doi:10.1158/2767-9764.CRC-23-0087)
Supplement: Supplementary Table S7 — Median survival time of PC patients treated with surgical resection and chemotherapy after classification into serum CA19-9 high (≥37 U / ml) and low (<37 U / ml) groups. [file crc-23-0087-s12.pdf]

Supplementary Table S7

Median survival time (Month)

	CA19-9 < 37 U/mL	CA19-9 ≧ 37 U/mL	P-value
Surgical resection	57.8	26.5	0.002
Chemotherapy	8.7	13.9	0.370

Median survival time of PC patients treated with surgical resection and chemotherapy after classification into serum CA19-9 high (≥37 U / ml) and low (<37 U / ml) groups.
