# Supplementary material for: EphA2 Proteolytic Fragment as a Sensitive Diagnostic Biomarker for Very Early-stage Pancreatic Ductal Carcinoma
Source: Cancer Res Commun. 2023 Sep 15;3(9):1862–74. doi: 10.1158/2767-9764.CRC-23-0087 (PMC10503484; doi:10.1158/2767-9764.CRC-23-0087)
Supplement: Supplementary Table S8 — Median survival time of PC patients treated with gemcitabine and nab-paclitaxel (GnP) and modified FOLFIRINOX (oxaliplatin, irinotecan, and 5-fluorouracil, mFFX) after classification into serum EphA2-NF high (≥50 pg / ml) and low (<50 pg / ml) groups. [file crc-23-0087-s13.pdf]

Supplementary Table S8

Median survival time (Month)

Treatment	EphA2-NF <50 pg/mL (n)	EphA2-NF ≧50 pg/mL (n)	P value
GnP	17.2 (56)	9.5 (151)	<0.001
mFFX	16.3 (9)	15.3 (16)	0.940

Median survival time of PC patients treated with gemcitabine and nab-paclitaxel (GnP) and modified FOLFIRINOX (oxaliplatin, irinotecan, and 5-fluorouracil, mFFX) after classification into serum EphA2-NF high (≥50 pg / ml) and low (<50 pg / ml) groups.
