# Supplementary material for: EphA2 Proteolytic Fragment as a Sensitive Diagnostic Biomarker for Very Early-stage Pancreatic Ductal Carcinoma
Source: Cancer Res Commun. 2023 Sep 15;3(9):1862–74. doi: 10.1158/2767-9764.CRC-23-0087 (PMC10503484; doi:10.1158/2767-9764.CRC-23-0087)
Supplement: Supplementary Table S9 — Median survival time of PC patients treated with GnP (G) and mFFX (F) classified into CA19-9 high (≥37 U / ml) and low (<37 U / ml) groups. [file crc-23-0087-s14.pdf]

Supplementary Table S9

Median survival time (Month)

Treatment	CA-19-9 <37 U / mL (n)	CA19-9 ≧37 U / mL (n)	P value
GnP	8.1 (39)	13.7 (168)	0.695
mFFX	17.5 (5)	15.3 (20)	0.525

Median survival time of PC patients treated with GnP (G) and mFFX (F) classified into CA19-9 high (≥37 U / ml) and low (<37 U / ml) groups.
