# Supplementary material for: EphA2 Proteolytic Fragment as a Sensitive Diagnostic Biomarker for Very Early-stage Pancreatic Ductal Carcinoma
Source: Cancer Res Commun. 2023 Sep 15;3(9):1862–74. doi: 10.1158/2767-9764.CRC-23-0087 (PMC10503484; doi:10.1158/2767-9764.CRC-23-0087)
Supplement: Supplementary Table S10 — Statistical analysis of the Allred score ratio (ASR) of IPMN with and without PC. The chi-square test was performed. [file crc-23-0087-s15.pdf]

# Supplementary Table S10

| | **Coexistence of PC in IPMN** | | ***p* value** |
|---|---|---|---|
| | **+** | **-** | |
| **ASR≥1** | 0 | 8 | ***p* = 0.03** |
| **ASR<1** | 19 | 31 | |

Statistical analysis of the Allred score ratio (ASR) of IPMN with and without PC. The chi-square test was performed.
